# Chronic Urinary Retention due to Fowler's Syndrome

**DOI:** 10.1055/s-0038-1672147

**Published:** 2018-10-18

**Authors:** Jan Trachta, Johann Wachter, Jan Kriz

**Affiliations:** 1 Department of Paediatric Surgery and Urology, Motol University Hospital, Charles University in Prague, Prague, Czech Republic; 2 Department of Urology, Kaiser-Franz-Josef Spital, Wien, Austria

**Keywords:** urinary retention, Fowler's syndrome, sacral neuromodulation

## Abstract

Fowler's syndrome (FS) is a rare cause of chronic urinary retention in teenage girls and young women. We present a case of a 14-year-old girl who presented at our hospital 2 weeks after uncomplicated laparoscopic appendectomy. The girl complained of reduced urinary frequency and prolonged micturition time. Following an acute cystitis 2 months later, she completely lost her ability to void. A comprehensive set of investigations to assess the cause of her urinary retention including a cerebral and spinal magnetic resonance imaging (MRI), and videourodynamics were performed. The diagnostic workup revealed polycystic ovaries and an asensitive and hypotonic bladder with capacity up to 1200 mL and high maximum urethral pressure of 120 cm of water. She did not tolerate clean intermittent catheterization; therefore, a suprapubic catheter was placed. Under this treatment, she suffered recurrent urinary tract infections. Two years later, she was diagnosed with FS on the basis of the medical history, clinical symptoms, and urodynamic findings. Finally, the implantation of a S3 neurostimulator restored her ability to void.

## Introduction

In 1988, Clare J. Fowler first described a syndrome which occurs in teenage girls and young women. It is characterized by chronic urinary retention with the history of general anesthesia for varying reasons, prolonged use of opiates, or delivery. 1 In around 50% of the patients, there is an association with polycystic ovaries or endometriosis. The functional retention is caused by external urethral sphincter (EUS) spasm due to vicious circle of autonomous excitation of sphincter muscle cells and failure to relax. EUS hyperactivity is followed by an asensitive and hypotonic bladder with permanent loss of the ability to void. 2

## Case Report

A 14-year-old previously healthy girl presented 2 weeks after an uncomplicated laparoscopic appendectomy for non-perforated acute appendicitis in a regional hospital. The girl complained of gradually reducing urinary frequency to twice per day and prolonged hesitancy. The micturition stream was initially weak and slow before becoming interrupted. Straining did not produce stronger urinary stream. She had never suffered from urinary tract infections (UTIs) or constipation and opened her bowels daily.

Following an episode of acute cystitis 2 months later, she completely lost her ability to void. She was put on indwelling Foley urinary catheter, and her cystitis was successfully treated with antibiotics. After every attempt to remove the urinary catheter, she had to be catheterized again with 300 to 1200 mL of urine volume registered. She noted loss of urge to urinate and felt only dull pain in suprapubic region and right iliac fossa on extreme bladder distention. The girl was kept on indwelling urethral urinary catheter and referred to a tertiary center to determine the etiology of her urinary retention.

She was examined with normal clinical findings and no obvious pathology on abdominal and pelvic ultrasound scan (USS). A pediatric neurologist found nothing abnormal, and magnetic resonance imaging (MRI) of the brain and spine, electromyography (EMG) of the lower extremity, somatosensory-evoked potentials (SEP) of tibial nerve, electroencephalogram (EEG), and lumbar puncture were with no pathology. On USS, the gynecologist described multiple follicular cysts on ovaries bilaterally and found no pathology explaining her urinary retention.

**Fig. 1:**
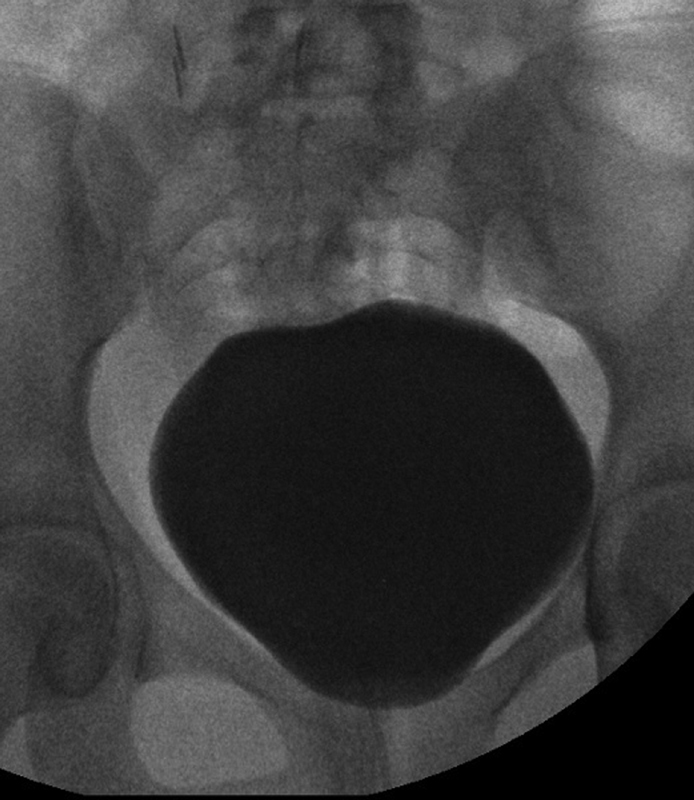
Normal looking bladder on videourodynamic study.

Our pediatric urologist performed an examination under general anesthesia including a free calibration of the urethra up to 26F followed by normal findings on cystoscopy. Videourodynamic study (VUDS) showed an asensitive and hypotonic bladder. The bladder filling had to be stopped at 360 mL due to the patient's discomfort. Maximum intravesical pressure achieved 11 cmH _2_ O. When pulling the urodynamic catheter out of the bladder manually, the maximum urethral pressure measured was 120 cmH _2_ O. On vesicocystourethrogram (VCUG), there was no vesicoureteral reflux, a smooth bladder wall and closed bladder neck ( Fig. 1 ).

Psychologic and psychiatric evaluation identified no major problem. During the following 2 years of repeated admissions to several regional and university hospitals, many of the tests described above were repeated, including an MRI of brain and spine with identical conclusions.

Clean intermittent catheterization (CIC) was recommended to the patient. However, because of poor tolerance of CIC due to frequent macroscopic hematuria and pain, a suprapubic catheter was placed. Thereafter, she suffered recurrent symptomatic afebrile UTIs caused by multi-resistant bacterial strains, e.g., *Klebsiella* , *Pseudomonas* , or *Escherichia* . Finally, after 2 years, based on the history, symptoms, and urodynamic findings, she was diagnosed with Fowler's syndrome (FS).

**Fig. 2:**
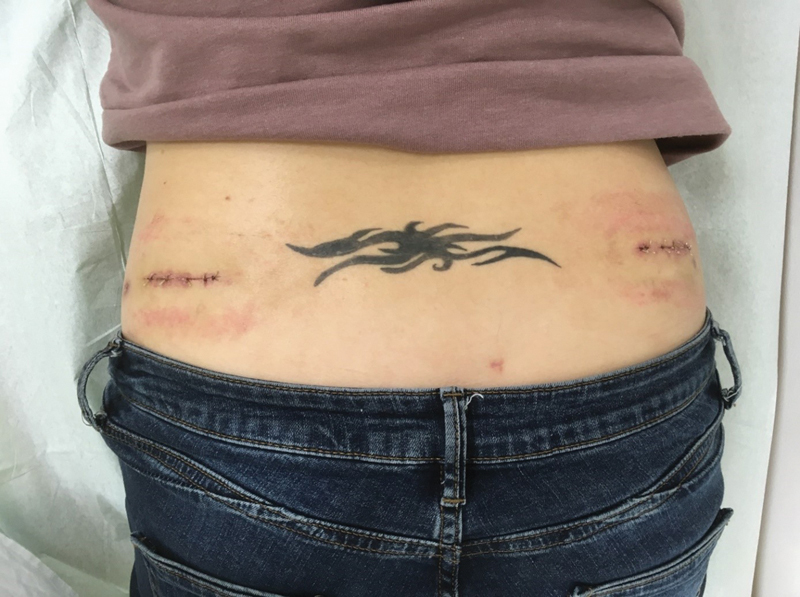
Wounds after bilateral S3 neurostimulator implantation.

For the treatment of FS, the patient was indicated for S3 neurostimulation. The implantation of two Medtronic S3 neurostimulators, type Interstim II, bilaterally in the upper gluteal region was performed under general anesthesia in two phases. The first phase was a transcutaneous implantation of the electrodes into S3 foramina and their connection to externalized neurostimulators. The first procedure took 30 minutes. As the patient restored her voiding completely back to normal when switching on the neurostimulators and experienced no side effects, she could undergo the second phase 4 weeks later—permanent subcutaneous implantation of the neurostimulators ( Fig. 2 ). The second procedure took 15 minutes under general anesthesia.

**Fig. 3:**
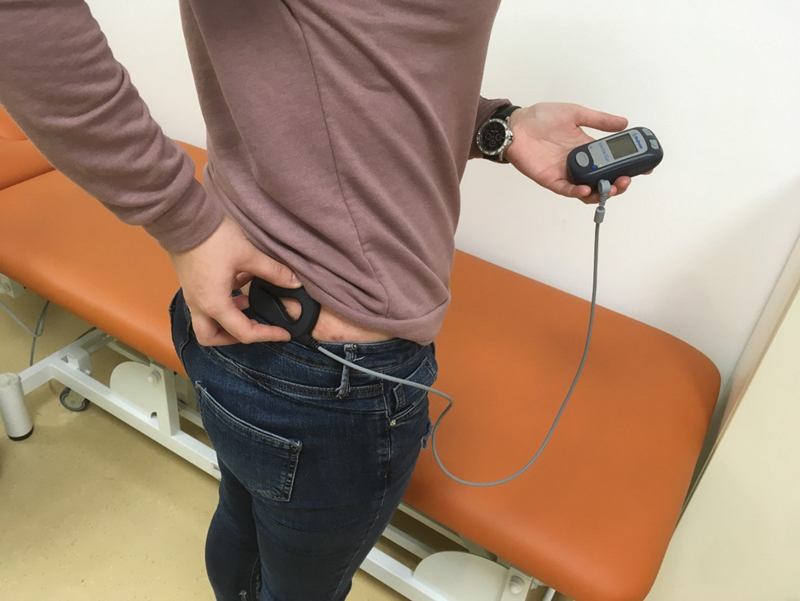
Transcutaneous remote control of current intensity of the S3 neurostimulators.

With a transcutaneous remote control, she was able to modify the intensity of stimulating current to avoid any discomfort ( Fig. 3 ). On the last follow-up, 4 months after the implantation, she voided four to six times per day with post-void residuals up to 50 mL on USS. Unfortunately, she suffered two prolonged episodes of burning on micturition even after the operation. On both occasions, she was diagnosed with acute cystitis by *E. coli* 107 that was treated with antibiotics after sensitivity testing.

## Discussion

Since the first publication on FS in 1988, it is not clear if FS is an extreme form of dysfunctional voiding with EUS hyperactivity followed by hypotonic bladder or distinct clinical unit. 2 However, every clinician facing unexplained urinary retention in teenage girls and young women should consider this diagnosis as there is a well-known treatment for FS. S3 sacral neuromodulation has a success rate of around 70% of full restoration of spontaneous voiding. 2

FS is described only in hormonally active women between menarche and menopause, never in males, with a peak incidence between 20 and 30 years of age. 3 A possible, however, not proven, hormonally dependent etiology is supported by the high association with polycystic ovaries or endometriosis in up to 50% of the patients compared with 20 to 30% in healthy female population. 4

Pathognomonic clinical signs are unexplained urinary retention of more than 1 L of sterile urine on catheterization, an asensitive bladder with loss of urge to void, and no help of straining, poor tolerance of clean intermittent self-catheterization. Difficulties of self-catheterization are typically described as a sense of “something gripping” the catheter inside the bladder as it is being pulled out of the bladder. 3

The diagnosis should be made after excluding organic cause of urinary retention. 5 The differential diagnosis of chronic functional urinary retention includes dysfunctional voiding, primary bladder neck obstruction, idiopathic detrusor underactivity, and chronic intestinal pseudo-obstruction. 2

According to the International Children's Continence Society (ICCS), dysfunctional voiding is defined as an intermittent and/or fluctuating flow rate due to intermittent contractions of the peri-urethral striated or levator ani muscles during voiding in neurologically normal children. 6 Rarely, there are cases of dysfunctional voiding that end up with complete retention (previously called non-neurogenic neurogenic bladder or Hinman syndrome). However, these children have severe detrusor hypertrophy on USS, often with secondary dilatation of upper urinary tract and not atonic or hypotonic bladder. 7

By kinesiological EMG, Deindl et al were able to register the activity of EUS separately from the rest of the pelvic diaphragm during voiding. The authors described an abnormal hyperactivity of the whole diaphragm including levator ani muscles and external anal sphincter during dysfunctional voiding. In contrast, in FS, there was only isolated EUS hyperactivity. 8

However, Fowler et al described not only a hyperactivity of the EUS, but a very specific hyperactivity. 9 The diagnosis of FS remains challenging. By usage of concentric needle EMG electrodes, Fowler et al proved so called ephaptic inter muscle fibers signal transmission due to indirect electric fields. 1 9 They found massive waves of activity called complex repetitive discharges and decelerating bursts. These studies suggest that striated muscle fibers of EUS are activating each other via neighboring membranes in parallel waves (in a similar manner as heart striated muscle fibers) and not via normal synaptic transmission on neuromuscular junction. Such parallel signals keep the EUS activated in a vicious circle and the loss of ability to relax continues.

The fact that the only tool to diagnose FS reliably is concentric EMG needle electrode makes the diagnosis difficult to determine in every day practice. In our case, we did not have a concentric EMG needle electrode available as this is found only in very few research centers with special interest in neurourology. Beside the history and clinical features mentioned above, there is a possibility to measure volume of EUS transvaginally on USS by 7.5 Hz probe. This suggestion is based on the hypothesis that long-term hyperactivity of EUS leads to its hypertrophy and higher volume. However, this investigation is limited by the interoperator variability in EUS measurement. 2

FS can also be diagnosed by a VUDS. The main VUDS findings in FS are delayed or no sensation of urge with large bladder capacity exceeding expected bladder capacity for age. The patients are usually unable to void, and there is no increase in detrusor pressure on excessive filling. Fowler et al describe maximum urethral closure pressure over 100 cmH _2_ O in FS on urethral profilometry. 9 However, other researchers oppose that this is a finding in many patients with dysfunctional voiding and is not specific for FS. 2

The finding of a hypoactive detrusor above a hyperactive sphincter is explained by pathologically stressed procontinence reflex. A poorly relaxing EUS increases urethral afferent activity, which inhibits bladder afferent signaling leading to poor bladder sensation and detrusor underactivity. The most frequently applied treatment is S3 sacral neuromodulation that—according to functional MRI and positron emission tomography (PET) brain studies in FS patients—restores bladder afferent signaling via stimulation of S3 of sacral roots. The pontine micturition center plus periaqueductal gray responds by “forcing” the detrusor to work in regular manner. 10

Sacral neuromodulation is a procedure, which is done in two stages. During the first stage, a stimulating lead is inserted into S3 sacral foramen under local or short general anesthesia. After connection of the lead to a neurostimulator, there is a 2- to 4-week period of trial. In case of positive answer, the neurostimulator is implanted definitively subcutaneously in the upper quadrant of buttocks. Disadvantages of this treatment include a 30 to 45% complications rate, mostly due to a technical failure or dislocation of the lead within years after the procedure. 2

Our patient underwent extensive diagnostic workup in several hospitals. The VUDS was performed only once with a suboptimal quality. Therefore, we have to acknowledge that despite the history and characteristic clinical features, the diagnosis of FS was never reliably confirmed in our case. However, given the fact that the history, and clinical course were typical and the treatment by sacral neuromodulation was successful, we are still very confident that the girl did suffer from FS.

## Conclusion

FS is a life-long condition. Despite a peak incidence between 20 and 30 years of age, it can happen in pubertal and adolescent girls, often after general anesthesia or use of opiates. Without EMG concentric needle electrode, the diagnosis cannot be determined reliably; however, it should be considered in all cases of unexplained chronic urinary retention.

## Notes

New Insights and the Importance for a Pediatric Surgeon Unexplained chronic urinary retention after any surgery performed under general anesthesia in pubertal girls might happen due to Folwer's syndrome. There is an effective treatment of this extreme lower urinary tract dysfuntion–sacral neurostimulation.
